# Voltammetric Detection of Caffeine in Beverages at Nafion/Graphite Nanoplatelets Layer-by-Layer Films

**DOI:** 10.3390/nano9020221

**Published:** 2019-02-07

**Authors:** Sandra Hernandez-Aldave, Afshin Tarat, James D. McGettrick, Paolo Bertoncello

**Affiliations:** 1 Systems and Process Engineering Centre, College of Engineering, Bay Campus, Swansea University, Crwmlyn Burrows, Swansea SA1 8EN, UK; 900162@swansea.ac.uk; 2 Perpetuus Advanced Materials, Unit B1, Olympus Court, Millstream Way, Swansea Vale, Llansamlet, SA7 0AQ, UK; afshintarat@perpetuuscarbon.com; 3 SPECIFIC, College of Engineering, Bay Campus, Swansea University, Swansea SA1 8EN, UK; J.D.Mcgettrick@Swansea.ac.uk; 4 Centre for NanoHealth, Swansea University, Singleton Campus, Swansea SA2 8PP, UK

**Keywords:** Nafion, graphite nanoplatelets, caffeine, electrochemical sensors

## Abstract

We report for the first time a procedure in which Nafion/Graphite nanoplatelets (GNPs) thin films are fabricated using a modified layer-by-layer (LbL) method. The method consists of dipping a substrate (quartz and/or glassy carbon electrodes) into a composite solution made of Nafion and GNPs dissolved together in ethanol, followed by washing steps in water. This procedure allowed the fabrication of multilayer films of (Nafion/GNPs)_n_ by means of hydrogen bonding and hydrophobic‒hydrophobic interactions between Nafion, GNPs, and the corresponding solid substrate. The average thickness of each layer evaluated using profilometer corresponds to ca. 50 nm. The as-prepared Nafion/GNPs LbL films were characterized using various spectroscopic techniques such as X-ray photoelectron spectroscopy (XPS), energy-dispersive X-ray spectroscopy (EDS), FTIR, and optical microscopy. This characterization highlights the presence of oxygen functionalities that support a mechanism of self-assembly via hydrogen bonding interactions, along with hydrophobic interactions between the carbon groups of GNPs and the Teflon-like (carbon‒fluorine backbone) of Nafion. We showed that Nafion/GNPs LbL films can be deposited onto glassy carbon electrodes and utilized for the voltammetric detection of caffeine in beverages. The results showed that Nafion/GNPs LbL films can achieve a limit of detection for caffeine (LoD) of 0.032 μM and linear range between 20‒250 μM using differential pulse voltammetry, whereas, using cyclic voltammetry LoD and linear range were found to be 24 μM and 50‒5000 μM, respectively. Voltammetric detection of caffeine in beverages showed good agreement between the values found experimentally and those reported by the beverage producers. The values found are also in agreement with those obtained using a standard spectrophotometric method. The proposed method is appealing because it allows the fabrication of Nafion/GNPs thin films in a simple fashion using a single-step procedure, rather than using composite solutions with opposite electrostatic charge, and also allows the detection of caffeine in beverages without any pre-treatment or dilution of the real samples. The proposed method is characterized by a fast response time without apparent interference, and the results were competitive with those obtained with other materials reported in the literature.

## 1. Introduction

Electrochemical sensors have received widespread attention in the analytical sciences due to their high selectivity and sensitivity, along with fast response time, ease of fabrication and low cost [1]. So far, carbon-based materials (glassy carbon, carbon paste, and carbon nanotubes) are among the most popular materials utilized as electrode surfaces as a result of their robustness and stability in various solvents, and wide potential range [2,3]. In 2004, the discovery of graphene, a 2D structure carbon allotrope, by Novoselov and Geim further extended the range of applications of carbon in electrochemical sensing: large surface area, high electrical conductivity, high mechanical and chemical stabilities are among properties that have contributed to the success of such materials [4,5,6]. The fabrication of graphene has become a main challenge in academic research and commercial applications. Several methods have been developed to produce graphene, including mechanical graphite exfoliation [7], carbon nanotubes unzipping [8,9], chemical vapor deposition of methane gas (CVD) [10,11] and reduction of graphene oxide [12,13]. These methods have shown low reproducibility, with high costs associated with the synthesis processes. Among carbon nanomaterials, graphite nanoplatelets (GNPs) consist of a small stack of platelet-shaped graphene with an average thickness of ca. 5–10 nanometers and with varying sizes up to 50 microns. GNPs can be produced in large quantities and at affordable cost; hence, they are very attractive from a commercial point of view. However, GNPs require correct functionalization in order to achieve the desired selectivity. One of the issues associated with the use of carbon nanomaterials in sensing is the difficulty of obtaining a good dispersion in aqueous solvents. One of the methods to improve dispersion is mixing GNPs with surfactants or polymers [14]. In this respect, polymers are preferable to surfactants because, in helping the dispersion, especially in benign solvents such as alcohols and water, they also confer on the composite material the physicochemical properties associated with the polymer. An example of this is Nafion, a sulfonic-based ionomer with cation exchange properties that is widely used in electroanalysis to preconcentrate positively charged species while repelling anions [15]. Typically, carbon nanomaterial‒polymer composites are deposited by drop casting their respective solutions on electrode surfaces to form micrometer-thick films or by spin coating to obtain thin films (sub-micrometer thick) [16]. A procedure to fabricate ultra-thin ionomer films has been previously developed by Bertoncello et al. [17]. The technique used the Langmuir‒Schafer method with the possibility of depositing a monolayer of the ionomer up to a few nm thick. Then, the as-prepared ionomer films were utilized for a variety of sensing applications. However, the Langmuir‒Schaefer method is not suitable for the mass production of electrode devices. Therefore, alternative methods are needed to prepare large volumes of these devices in a simple and affordable fashion. An attractive method is represented by the in situ (or electrostatic) layer-by-layer (LbL) method, developed in 1991 by Decher et al. [18]. This method is widely utilized for the preparation of thin films with controlled thickness, morphology, and internal organization on solid substrates. The LbL thin films are prepared via attractive forces of macromolecular components such as electrostatic forces, hydrogen bonding, and other intermolecular interactions [19,20]. The main advantages of this method are the reproducibility and scalability, making this method potentially attractive for commercial applications. In 2010 Lu et al. developed a method of self-assembling GNPs from stable suspensions of graphite nanoplatelets with polyelectrolytes (PEs) [14]. By using PEs with opposite charges, they were able to fabricate LbL thin films of GNPs composites, avoiding agglomeration and preserving the conductivity properties of GNPs. Keeping in mind the aforementioned work of Lu et al., in this paper we describe for the first time a method of fabricating Nafion/GNPs composite thin films using a modified LbL method. The oxygen functionalities available in the graphite nanoplatelets on the edges and on the basal planes of the flakes make possible interactions with the sulfonic groups of the ionomer via hydrogen bonding along with hydrophobic‒hydrophobic interactions between the carbon groups of graphite and the Teflon-like structure (carbon‒fluorine backbone) hydrocarbon structure of Nafion. The effect of the concentrations of GNPs and Nafion polymer in the film was analysed by tuning their concentrations in the solution. The electrochemical characterization of the as-prepared Nafion/GNPs LbL films showed the possibility of incorporating positively charged hexammine ruthenium chloride as a redox probe. As a result of this preconcentration ability to detect cations, we tested the Nafion/GNPs composite films for the detection of caffeine, a natural alkaloid widely present in food such as chocolate, coffee, soft drinks and tea. Detection of caffeine is not only important because of its widespread presence in beverages but also for its clinical relevance as a stimulant to the central nervous system and in the preparation of various drugs [21]. Caffeine is commonly detected using spectrophotometric methods [22]; however, electrochemical methods are more advantageous due to the simplicity of the apparatus and ease of detection. The method presented herein is appealing for several reasons, primarily the possibility of fabricating Nafion/GNPs films in an automated fashion and without the need for electrolytes of the opposite charge. In doing so, the procedure of film fabrication is greatly simplified and potentially compatible with mass production of sensor devices; secondly, the as-prepared Nafion/GNPs LbL films showed good electrochemical properties in terms of the limit of detection, sensitivity and linearity of range for caffeine detection. The values obtained for LoD are competitive with other systems utilized for the detection of caffeine reported in literature. More importantly, the as-prepared Nafion/GNPs LbL films allowed detection of caffeine in a wide linear range without any pre-treatment of dilution of samples. These characteristics make this method suitable for fast detection of caffeine in the beverage industry, where caffeine is added at relatively high concentrations.

## 2. Materials and Methods 

### 2.1. Reagents and Solutions 

Graphite nanoplatelets (GNPs) (surface0modified friable nano-graphite) were obtained from Perpetuus Carbon Technologies Ltd (Swansea, UK). GNPs were synthesized by employing dielectric barrier discharge (DBD) plasma with various working gases [23]. Nafion perfluorinated resin solution (5 wt % in lower aliphatic alcohols and containing 15‒20% water) was purchased from Sigma-Aldrich (Gillingham, Dorset, UK). Standard caffeine powder (Reagent Plus grade) was purchased from Sigma-Aldrich. NaCl (99.5%) and ethanol (absolute HPLC grade) were purchased from Acros Organics (Geel, Belgium). Aqueous solutions of NaCl at pH 2 were prepared by adjusting the as-prepared solutions of NaCl with aliquots of 0.5 M HCl solutions. All aqueous solutions were prepared from doubly distilled Milli-Q reagent water (Millipore Corp., Burlington, MA, USA) with a resistivity of 18 MΩ·cm at 25 °C.

### 2.2. Apparatus

Optical microscopy imaging was carried out using a Sarfus mapping nanoscale Zeiss microscope (Nanolane, Le Mans, France). FTIR measurements were performed using a Perkin Elmer, (Waltham, MA, USA). Spectrum Two FT-IR, mounted with a Diamond Crystal in attenuated total reflectance (ATR) mode. The resolution was 2 cm^−1^, with an accumulation of four scans per spectrum. A background scan was run before any acquisition experiment. The energy-dispersive X-ray spectra (EDS) were acquired using a JEOL 7800F (Tokyo, Japan) scanning electron microscope that was utilized for qualitative and quantitative analysis of the sample composition. The LED detector was used at 5 kV acceleration voltage. The count limit was 500,000. X-ray photoelectron (XPS) spectra were recorded using a Kratos Axis Ultra DLD photoelectron spectrometer (Kratos Analytical, Shimadzu Corporation, Kyoto, Japan) having a monochromatic Al-K_ᾳ_ X-ray source. XPS data analysis was performed using CasaXPS Version 2.3.17dev6.4k using the Kratos sensitivity factor library. The base model used to fit the XPS spectra is outlined elsewhere [24] and the d-parameter was also calculated based on the C LMM Auger [25]. The UV-vis spectra were made using a double Beam Hitachi Spectrophotometer U-2900/2910. The zeta-potentials of the GNPs solutions were measured using a Zetasizer (Nano-ZS, Malvern Instruments, Malvern, UK). Zeta-potential measurements were performed at room temperature in solutions at different pH values. Cyclic voltammetry measurements were performed using a CH Instrument (Bee Cave, TX, USA) Model 705 electrochemical work station using a conventional three-electrode cell. Differential pulse voltammetry measurements were performed using a CH Instrument Model 760 electrochemical work station. A rectangular Sigradur G-glassy carbon electrode plates (size 30 × 7 × 1 mm) were purchased from HTW (Thierhaupten, Germany). The electrical contact between the rectangular Sigradur G – glassy carbon electrode and the crocodile connection with the potentiostat was made using metallic self-closing tweezers (Dumont, Hatfield, PA, USA). The glassy carbon electrodes were first cleaned with methanol and then polished for 5 min with different grades of alumina powder (1 and 0.3 µm, respectively). Several CVs on GCE were performed in 0.1 M sulfuric acid to eliminate any residue from the previous experiments. Finally, a further step of polishing of the GCEs was performed using a 0.05 µm alumina powder. The glassy carbon plate was used as the working electrode and a platinum wire as the counter electrode. All potentials are quoted versus the Ag/AgCl reference electrode and all the measurements are recorded at room temperature. DPV scans were carried out as follows: increment 0.004 V, amplitude 0.05 V, pulse width 0.05 s, sampling width 0.0167 s, pulse period 0.5 s, *E*_initial_ 1 V; *E*_final_ 1.5 V.

### 2.3. Preparation of Nafion/GNPs Composite Solutions

Nafion/GNPs composite solutions were prepared by the addition of the corresponding concentration of Nafion into the GNPs dispersion in ethanol. For example, Nafion 0.5 wt % and GNPs 0.5 wt % composite solutions were made using the following procedure: 1 g of GNPs were dispersed in 100 mL of ethanol to obtain GNPs 1 wt %. These as-prepared GNPs solutions in ethanol were sonicated for 30 min in order to obtain uniform dispersion of the GNPs. Separately, Nafion 1 wt % solution was prepared by dissolving 10 mL of the commercial 5 wt % Nafion perfluorinated resin in 40 mL ethanol. The as-prepared Nafion 1 wt % was sonicated for 30 min. Finally, 20 mL of the Nafion 1 wt % were added to 20 mL of the solution containing GNPs 1 wt % and the composite was sonicated for 30 min. The as-prepared Nafion/GNPs composite solution is well dispersed, as documented in the video in the Electronic Supporting Information (ESI), Figure S1, which highlights the LbL procedure.

### 2.4. Preparation of Nafion/GNP LbL Films 

Prior to the deposition of Nafion/GNPs films for UV-visible characterization, the quartz slides were cleaned using piranha solution (3:1 H_2_SO_4_ /H_2_O_2_) for 30 min. Be aware that the solution produces a considerable amount of heat and that hydrogen peroxide must be added drop to drop with care inside a fume hood. This process allows the formation of hydroxyl functionalities in the surface of the substrate [26,27]. The cleaned substrates were thoroughly rinsed with DI water and dried with compressed air. The LbL assembly of Nafion/GNPs was done using an automated dip coater (Multi Vessel small KSV NIMA, Manchester, UK). The formation of Nafion/GNPs films consists of cyclic repetitions of two steps: (1) formation of the LbL layer by immersion of the substrate in the Nafion/GNPs composite; (2) washing the as-prepared LbL film in deionized water. The optimum immersion and withdrawal speed was selected as 50 mm/min. The films were dried at room temperature for 2 min. The film formation procedure of the LbL film is reported in S1. Multilayers of Nafion/GNPs, and defined as (Nafion/GNP)*_n_*, with *n* = number of layers, were performed by repeating the deposition and washing cycles until the desired number of layers (*n*) was achieved. 

### 2.5. Detection of Caffeine in Real Samples 

Nafion/GNPs LbL films were tested in commercial samples of Coca-Cola and energy drink (Power Energy Shot). Detection of caffeine was performed in samples of these drinks, which were used as received. Prior to measurement, the samples were degassed by vigorous stirring for 5 min to eliminate fizzing from the drinks. Then 0.1 M NaCl was added in the commercial drinks as a supporting electrolyte. The pH was determined using a HI-2002 Edge (Leighton Buzzard, UK) pH meter. The pH values of the fizzy beverage samples were determined to be 2.3 and 2.7 for Coca-Cola and the power energy shot, respectively.

## 3. Results

### 3.1. Morphology and Structure of Multilayer Nafion/GNPs Films

Typically, LbL films are fabricated by alternately dipping the substrate into solutions having opposite charges [28]. In this work we developed a different strategy of deposition in which the polymer and the graphite nanoplatelets are mixed together to form a homogenous composite solution. Hence, LbL multilayers films are obtained by immersing the substrate in the Nafion/GNPs composite solution, followed by washing in DI water. Therefore, only one immersion step is needed, followed by a washing step using DI water. There are two advantages to using such a method: (1), the addition of Nafion in the solution prevents the aggregation of the GNPs, giving good dispersion even in water; (2) the multilayer assembly is faster since it requires fewer steps, as the LbL layers are formed using the same composite solution in each deposition cycle.

**Figure 1:**
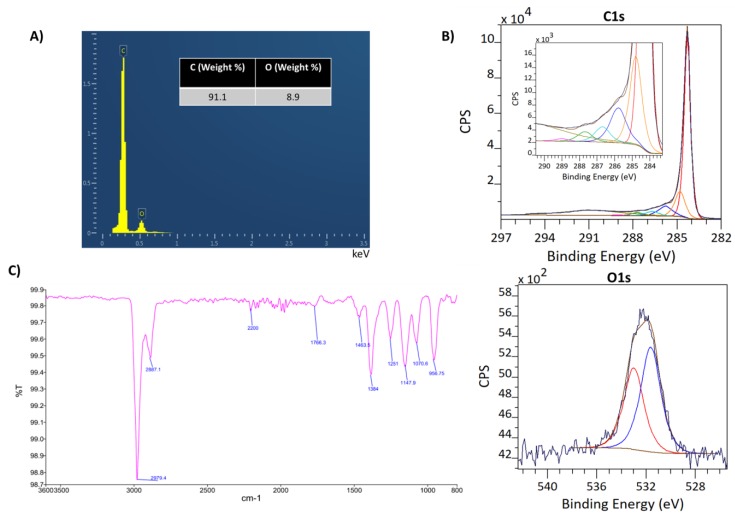
(**A**) Energy-dispersive X-ray spectra (EDS) of GNPs powder; (**B**) X-ray photoelectron emission spectroscopy of the C1s core level for GNPs powder. Inset: the magnification of the carbon components. Below the O1s core level for the GNPs powder. (**C**) FTIR spectra of GNPs powder.

Firstly, we characterized the GNPs powder using EDS, XPS, and FTIR to prove the presence of oxygen. Energy-dispersive X-ray spectroscopy (EDS) was used to determine the chemical composition of the graphite nanoplatelet powder. Figure 1a shows the EDS peaks corresponding to carbon and oxygen. The weight ratios are 91.1% and 8.9% for carbon and oxygen, respectively. The oxygen speciation was studied by means of XPS measurements. Figure 1b shows the C1s core-level XPS spectra of graphite nanoplatelets. The spectra show an intense peak at 284.3 eV with the distinctive asymmetric tail indicative of sp^2^ carbons, and representing the non-oxygenated graphene structure. Additionally analysis of the d-parameter of the carbon LMM Auger yields a high d-parameter of 23, indicating a high degree of sp^2^ hybridization and graphite-like material. The second major component at 284.8 eV is related to sp^3^ carbons [29]. Following a symmetrical peak model for the sp^2^ carbon, the spectrum shows several smaller peaks at 285.8 eV, which are attributed to the presence of hydroxyl (C‒OH) or epoxy (C‒O) functional groups, and a peak at 286.7 eV attributed to the C‒O‒C bonds. The XPS spectrum evidences additional peaks with lower intensity at 287.7 and 289.0 eV, which are associated with carbonyl (C=O) and carboxyl (O‒C=O) functional groups, respectively [30]. These functional groups are normally located at the edges of the nanoplatelet sheets, whereas the epoxy groups create defects localized at the basal planes of the flakes. The peak observed at higher binding energy (290.8 eV) corresponds to π–π* shake up satellite, indicative of the presence of extended aromatic rings. The O1s core-level spectrum for the GNPs is shown in Figure 1b (below) and was de-convoluted into two individual segments. The higher intensity peak at 531.7 eV is related to the single bond of oxygen to carbon (C‒O). The second peak at 533.0 eV is assigned to the double bonding of oxygen to an aromatic carbon (C=O) [31]. These results suggest a high concentration of epoxy functionalization in the basal plane of the graphene structure. The FTIR spectroscopy is presented in Figure 1c, which confirms the presence of the different functional groups as seen using XPS. FTIR spectroscopy is presented in Figure 1c, which confirms the presence of the different functional groups, as seen using XPS. The FTIR spectra shows two intense absorption peaks at 2979 and 2887 cm^−1^ related to the asymmetric and symmetric C‒H stretching modes, respectively, of alkyl moieties [29]. Peaks at 1463 and 1384 cm^−1^ arise due to aliphatic C‒H bending vibrations for methyl and methylene groups [32]. The peaks located at 1251 and 956 cm^−1^ are ascribed to the stretching vibrations of the epoxy groups (C‒O‒C) [33,34,35]. The peaks at 1147 and 1070 cm^−1^ are attributed to C‒O vibrational mode for alkoxy groups [36,37,38]. The small peak at 1766 cm^−1^ corresponds to the C=O stretching vibration mode [39].

The interactions of GNPs with Nafion are likely to occur via synergistic hydrogen bonding between the carboxylate and epoxy groups available at the GNPs surface with the sulfonic groups of Nafion, in addition to hydrophobic‒hydrophobic interactions between the carbon groups of GNPs with the fluoroalkyl chain of Nafion [29].

**Scheme 1:**
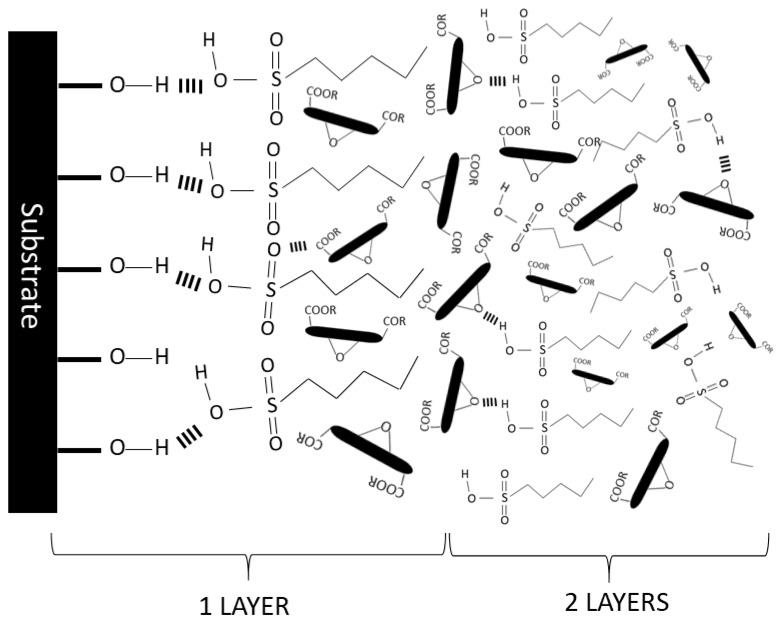
Schematic of Nafion/GNPs LbL assembly.

Nafion is a copolymer comprising a hydrophobic part that corresponds to the fluorocarbon backbone and a hydrophilic part constituted of sulfonic groups. The latter confer Nafion cation exchange properties, along with superacidic characteristics [15]. The carboxylate and epoxy groups in the GNPs allow the formation of hydrogen bonding with the sulfonic groups of Nafion. The long fluorocarbon chains in the Nafion generate a hydrophobic environment in the molecule, whereas GNPs with lower quantities of oxygen groups are more hydrophobic and mainly interact with the Nafion backbone. Hence, the formation of LbL layers occurs via a synergistic combination of hydrogen bonding and hydrophobic interactions. Both these interactions are crucial for the LbL film formation. It is worth mentioning that Nafion/GNPs LbL films are destroyed when the films are immersed into a solution of NaOH at pH 10, as at this pH value, hydrogen bonding interactions are destroyed. The importance of hydrophobic interactions during the formation of multilayers has been reported in the past, as polymers with opposite charges are unable to form films via LbL due to thermodynamic instability. A molecule must have properly balanced hydrophilic and hydrophobic properties in order to self-assemble. If a molecule is too hydrophilic, small and highly charged, it will not form a thermodynamically stable complex [40,41,42,43,44,45]. Based on our findings, the proposed mechanism of self-assembling is shown in Scheme 1. 

### 3.2. Effect of Graphite Nanoplatelets and Nafion Concentration in the LbL Film Formation

**Figure 2:**
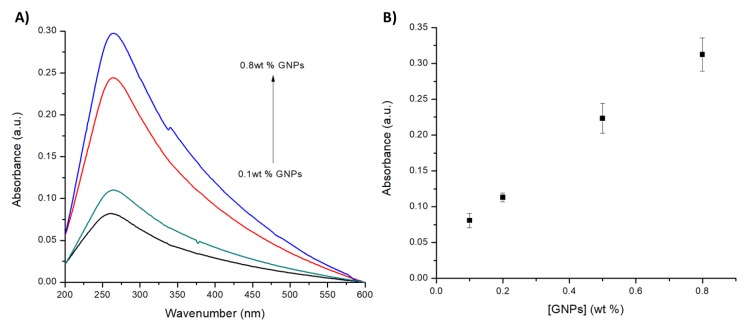
UV-vis spectra of five Nafion/GNPs LbL layers at different concentrations of GNPs: (**A**) absorbance curve at different GNPs concentrations; (**B**) UV-visible plot of five Nafion/GNPs LbL layers with the concentration of GNPs.

The effect of the GNPs and Nafion in the LbL film formation was analysed by tuning the Nafion/GNP concentrations. In this respect, different concentrations of GNPs were used (namely 0.1, 0.2, 0.5 and 0.8 wt %). The ratio of Nafion/GNPs was kept constant at 1. The increase of the GNPs concentration in the film was analysed using UV-visible absorption spectroscopy. Figure 2 depicts the UV-vis spectra of 5 LbL layers obtained at different GNPs concentrations, with the appearance of a band at 265 nm corresponding to a π‒π* transitions of the sp^2^ C=C bonds, typical of reduced graphene oxide. The increasing number of π-conjugations present in the reduced graphene, in comparison with the graphene oxide (GO), means that it requires less energy for the π‒π* transition, which normally is found at 235 nm, shifting to the longer wavelength region [46]. Nafion does not show any absorbance peak at such value. The absorbance increases with the concentration of GNPs; however, at concentrations of 0.8 wt % it is only possible to deposit up to 7 LbL films. At *n* > 7 LbL layers and 0.8 wt % concentration, the films tend to break down, as shown in Figure S2. We explain this effect due to the electrostatic repulsions between the negatively charged graphite nanoplatelets in the film (zeta potential: ‒40 mV). The use of a high concentration of GNPs (0.8 wt %) leads to a large number of graphite nanoplatelets superimposed on the film, where the strong electrostatic interactions between the nanoplatelets could break the thermodynamic stability and consequently break down the film.

**Figure 3:**
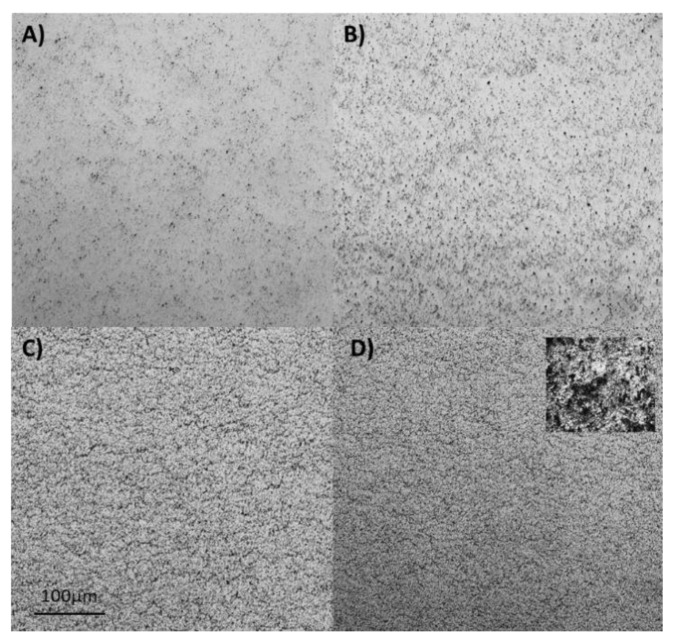
Optical microscopy imaging of five Nafion/GNPs LbL layers corresponding to (**A**) 0.1 wt % GNPs; (**B**) 0.2 wt % GNPs; (**C**) 0.5 wt % GNPs; and (**D**) 0.8 wt % GNPs. The inset shows a magnified image of the LbL films obtained at 0.8 wt % GNPs.

The morphology of the as-prepared LbL films obtained at different GNPs concentration was evaluated using optical microscopy. Figure 3 shows the microscopy patterns of Nafion/GNPs at different concentrations of GNPs. The pattern observed at 0.5 and 0.8 wt % evidences a good dispersions of the GNPs along the film, without the formation of clusters or agglomerated. However, as previously stated, we observed that for high number of layers (*n* > 7 layers) for 0.8 wt % the film starts to show the presence of GNP agglomerates and tends to break down. Therefore, for this work, we selected 0.5 wt % GNPs as the ideal concentration of graphene nanoplatelets for the LbL film formation. 

The effect of the concentration of Nafion in the LbL film formation was determined by keeping constant the concentration of GNPs at 0.5 wt % and by changing the concentration of Nafion resin from a minimum of 0 (no Nafion) to a maximum of 1.5 wt %. In the case of pristine GNPs (without the addition of Nafion) the LbL film formation is inhibited, showing only some random flakes attached to the substrate (see Figure S3). This response is expected as Nafion improves dispersion and prevents agglomeration of the GNPs, but also it contributes by providing available sites (sulfonic groups) to form hydrogen bonds with the hydroxylic groups of the quartz substrate, which in turn drives the LbL film formation (see Scheme 1). In the case of 0.1 wt % Nafion, we observed the formation of the LbL film formation as an indication of the importance of Nafion in the procedure of the film formation. A concentration of 1.5 wt % of Nafion shows a decrease in the absorbance value and the film formation becomes more difficult (figure not shown). We explain this effect by the excess of negative charge at high concentrations of Nafion, which contributes to the increase of electrostatic repulsion between the sulfonic groups of Nafion and carboxylated functionalities of GNPs. For this reason, the optimum concentration of Nafion was selected as 1 wt %. Based on the experimental evidence, we selected 1 wt % of Nafion and 0.5 wt % of GNPs as the optimum concentration for the LbL film formation.

**Figure 4:**
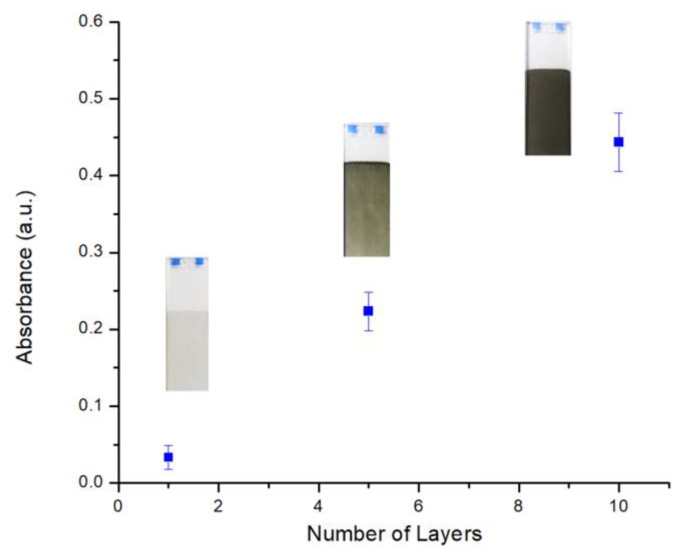
UV absorption of Nafion 1 wt %/GNPs 0.5 wt % with the number of layers at 265 nm.

Figure 4 shows the UV-Vis absorption spectra of Nafion/GNPs with a different number of layers deposited on quartz slides. The progressive increase of the absorption peak at 265 nm with the number of layers is clearly visible. The linear relationship between the absorbance and the number of layers evidences a reproducible and uniform LbL assembly process.

### 3.3. Electrochemical Characterization of Multilayer Nafion/GNPs Films

**Figure 5:**
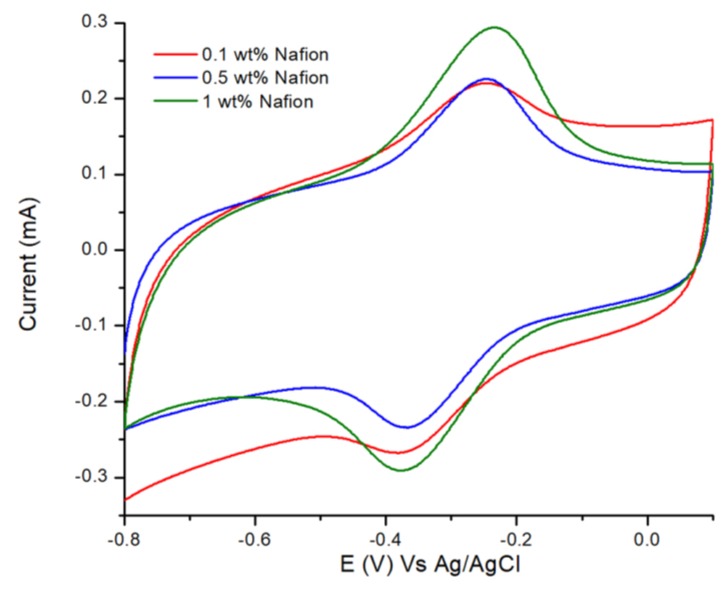
Cyclic voltammetry (CVs) of Nafion/GNPs 5 LbL films at different concentrations of Nafion (from 0.1 to 1 wt %) with GNP concentration at 0.5 wt %. The films were loaded in 5 mM Ru[(NH_3_)]_6_^3+^, and then transferred into 0.1 M NaCl supporting electrolyte; scan rate 50 mV s^−1^.

The electrochemical properties of Nafion/GNPs LbL films were tested using cyclic voltammetry. Hexammineruthenium (III) chloride was selected as the cationic redox probe in order to investigate the preconcentration properties of the as-prepared Nafion/GNPs LbL films. Nafion/GNPs LbL films were at first loaded in a 5 mM solution of Ru[(NH_3_)]_6_^3+^ for 10 minutes to achieve full preconcentration (Figure S4). Figure 5 shows the CVs of five Nafion/GNPs LbL films at different concentrations of Nafion previously loaded in 5 mM Ru[(NH_3_)]_6_^3+^ after transferred into 0.1 M NaCl as a supporting electrolyte. The CV shows the appearance of the typical redox peaks of the couple Ru[(NH_3_)_6_]^3+/2+^ at ‒0.23 V (oxidation) and ‒0.37 V (reduction) vs Ag/AgCl. The Nafion/GNPs LbL films confirm the ability of the composite material to incorporate and retain the redox mediator. The peak currents increase with Nafion concentration from Nafion 0.1 wt % to 1 wt %. For concentrations of Nafion higher than 1 wt % the peak current does not increase, hence we selected Nafion 1 wt % as this concentration provides a high peak current but also a better quality of Nafion/GNPs LbL films. 

**Figure 6:**
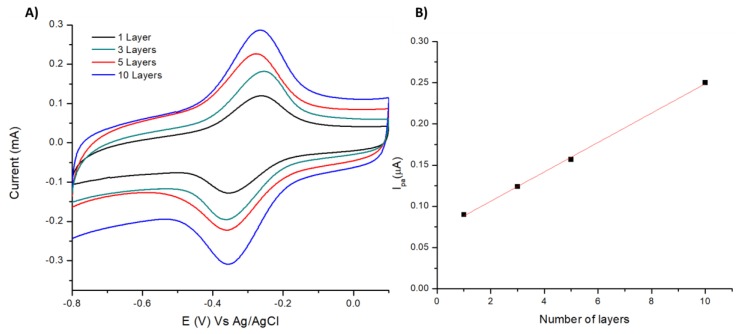
(**A**) CVs of Nafion/GNPs at different number of layers loaded in 5 mM Ru[(NH_3_)]_6_^3+^ after transferring in 0.1 M NaCl supporting electrolyte, scan rate 50mV s^−1^. (**B**) Plot of the oxidation currents vs. the number of layers (from 1 to 10 layers).

Figure 6 shows the CVs of different number of Nafion/GNPs LbL films, previously loaded in 5 mM solution of Ru[(NH_3_)]_6_^3+^ recorded after being transferred into 0.1 M NaCl supporting electrolyte. As expected, the peak currents increase with the number of layers, with a concomitant increase of the background current as a result of the higher active geometric area provided by the deposition of GNPs on GCE surface. For the purpose of experiments for the detection of caffeine, we selected five LbL layers, as a higher number of LbL layers does not improve sensitivity.

The active surface area of the electrode was determined by performing chronocoulometry experiments on bare GCE, 5 and 10 Nafion/GNPs LbL layers. The chronocoulometry was recorded in 5 mM Ru[(NH_3_)]_6_^3+^ with 0.1 M NaCl as the supporting electrolyte. Analysis of chronocoulometric data allowed us to draw the plot of the charge (Coulomb, *C*) vs. the time (*t*). These plots are shown in Figure S5; by plotting the charge (*C*) vs. the square root of time (*t*^1/2^), it is possible to find the active surface area by using the integrated Cottrell equation [47,48]:

(1) $$Q=\frac{2nFAD^{\frac{1}{2}}C_{0}t^{\frac{1}{2}}}{\pi^{\frac{1}{2}}},$$

where *n* is the number of electrons involved in the redox reaction, F is the Faraday constant, *A* is the geometric electrode area (cm^2^), *D* is the diffusion coefficient of the redox mediator and *C*_0_ is the concentration of the redox mediator in solution. In this case, using Ru[(NH_3_)]_6_^3+^ as a redox probe, with a diffusion coefficient value of 8.43 × 10^-6^ cm^2^·s^−1^, as reported in the literature [49], the area of bare GCE calculated by the electrochemical method was 1.303 cm^2^. Such a value is close to the geometric area of the bare electrode (1.26 cm^2^). The area of Nafion/GNPs obtained with the coulometric method for five and 10 LbL layers was much larger—1.645 cm^2^ and 1.702 for five and 10 LbL layers, respectively. This is not surprising taking into account that the LbL assembly process leads to an effective increment of the electrode surface (GNPs are randomly oriented on GCE electrodes), as evidenced by higher peak currents observed in Nafion/GNPs compared to bare GCEs.

The apparent diffusion coefficients for Nafion/GNPs were estimated using the procedure utilised for ultra-thin pristine Nafion films deposited using the Langmuir‒Schaefer method [50,51,52,53]. By integrating the area of the voltammetric peak at lower scan rate (10 mV), where exhaustive electrolysis of the redox mediator takes place under thin-layer-like conditions, it is possible to estimate the surface coverage. For this purpose we utilised the CV patterns of five Nafion/GNPs LbL films loaded in 5 mM Ru[(NH_3_)]_6_^3+^ and transferred them into 0.1 M NaCl supporting electrolyte (figure not shown). The CVs were recorded at a low scan rate (10 mV), where the peak to peak separation is 44 mV. The surface coverage, *Γ* is calculated accordingly to the equation:

(2) $$\Gamma =\frac{Q}{nFA},$$

where *Γ* is the surface coverage (mol cm^−2^), *Q* is the charge at low scan rate (C) and *n* is the number of electrons transferred in the redox reaction, F is the Faraday constant and *A* is the active electrode area (cm^2^) calculated using chronocoulometry. The concentration of Ru[(NH_3_)]_6_^3+^ inside of the film is given by the following equation:

(3) $$C_{0}^{*\;}=\frac{\Gamma }{\Phi },$$

where *C*_0_^*^ is the concentration of Ru[(NH_3_)]_6_^3+^ within the composite film and *Φ* is the thickness of the film (cm) calculated using profilometer. The apparent diffusion coefficient, *D*_app_, at 25 °C, was calculated using the Randles‒Sevcik equation [47]:

(4) $$I_{p}=\left(2.69\times 10^{5}\right)n^{\frac{3}{2}}AC_{0}^{*\;}v^{\frac{1}{2}}D_{\mathrm{app}}{}^{\frac{1}{2}},$$

where the *I_p_* is the maximum current (A) under diffusion-controlled conditions and *v* is the scan rate (V·s^−1^).

Noticeably, if the area of the electrode is kept constant during the voltammetric experiments, exact knowledge of the electrode has no influence on the estimation of *D*_app_. The results obtained (see Table 1) show that *D*_app_ values are on the order of 10^−11^/10^−10^ cm^2^·s^−1^, with values of concentration of Ru[(NH_3_)]_6_^3+^ inside the film on the order of 10^−2^/10^−1^ mol dm^−3^. These values are consistent despite being slightly larger than those reported for Nafion Langmuir‒Schaefer ultra-thin films. This trend is somewhat expected since the introduction of GNPs increases the porosity and roughness of the films, allowing faster diffusion of the redox mediator within the film when compared to Langmuir‒Schaefer films. However, it is worth noting that the values of *D*_app_ and *C*_0_* have to be taken as a rough estimation and assumed to have an intrinsic error, since Nafion swelling is well established when immersed in solution, and the values of thickness utilised for the calculations have been obtained in dry state (see Figure S6).

### 3.4. Detection of Caffeine

**Figure 7:**
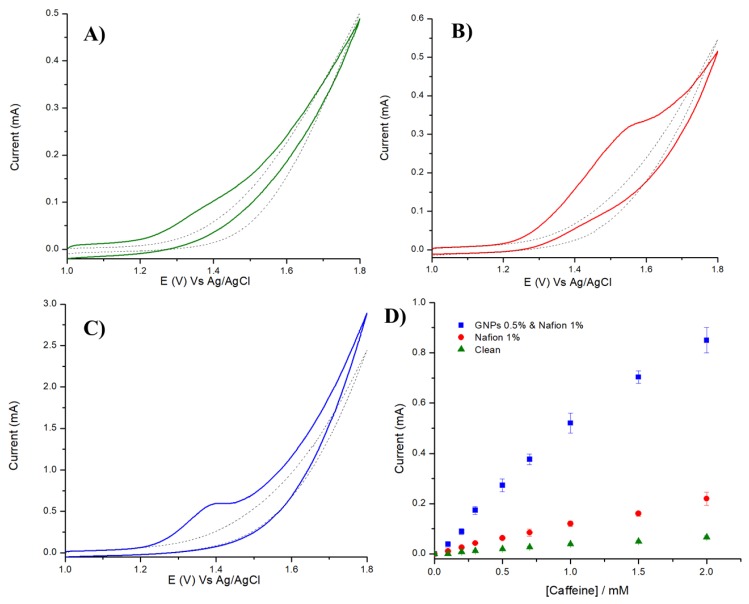
CVs of (**A**) Bare GCE, (**B**) Nafion, (**C**) Nafion 1 wt %/GNPs 0.5 wt % (five LbL layers) films in the presence of 0.5 mM caffeine, recorded in 0.1 M NaCl supporting electrolyte (pH 2); scan rate, 50 mV·s^−1^. (**D**) Plot of the oxidation currents vs. concentration of caffeine (from 0.1 to 2 mM) for bare GCE (green triangles), Nafion (red circles) and Nafion/GNPs (blue rectangles).

We utilised five Nafion/GNPs LbL layers to investigate the suitability of such composite for the detection of caffeine. The aim of the as-prepared sensor is the detection of caffeine in fizzy beverages without pre-treatment or dilution of the samples. The electrochemical behaviour of Nafion/GNPs (five LbL layers) in the presence of caffeine were investigated using cyclic voltammetry and differential pulse voltammetry. CV and DPV experiments were performed using 0.1 M NaCl as a supporting electrolyte solution. The pH was acidified to obtain pH 2 using HCl. Acidic pH is needed in order to improve the solubility of the caffeine [54], but also to mimic the natural pH of fizzy drinks (usually the pH is between 2 and 3). Figure 7 reports the voltammetric behaviour of bare GCE, Nafion, and a Nafion/GNPs composite recorded in 0.1 M NaCl, pH 2 containing 0.5 mM of caffeine. The CV shows the presence of an irreversible oxidation peak at 1.4, 1.5, and 1.37 V for bare GCE, 1% Nafion-coated film, and five LbL layers of Nafion/GNPs-coated electrodes, respectively. The peak observed for the bare GCE at 1.4 V shows a potential shift towards positive potential as the concentration of caffeine increases, which shifts up to ca. 1.8 V for the highest concentration of caffeine (see Figure S7). The fact that the irreversible oxidation of caffeine occurs at a less positive potential in the case of Nafion/GNPs LbL films is an indication that the composite coating facilitates the oxidation of caffeine. This also contributes to avoiding the potential interference from water oxidation that may occur at such potential. This effect has already been described by Zanardi et al. [55] and confirmed in one of our previous works [56].

Figure 7D reports the plot of the oxidation peak at different concentration of caffeine. The peak currents related to Nafion/GNPs are remarkably higher compared to the bare GCE (4-fold) and 1% Nafion (3-fold) coated film confirming the suitability of such material towards the detection of caffeine. This is because, at pH 2, caffeine is likely to be in its protonated form (see Figure S6) and, therefore, it is preconcentrated within the negatively charged Nafion/GNPs composite, giving rise to a higher peak current than the bare GCE. Detailed CVs of Nafion/GNPs are reported in Figure S8.

**Figure 8:**
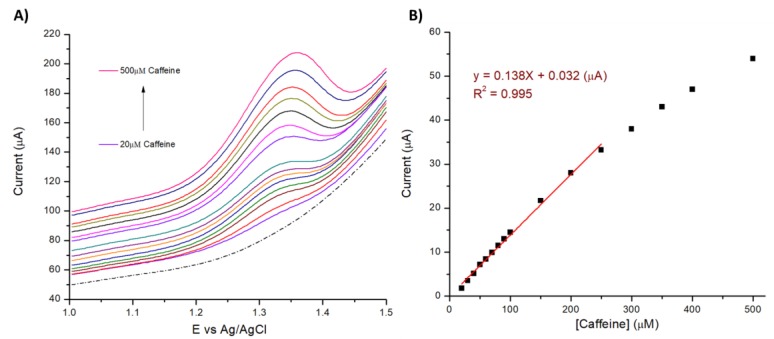
(**A**) DPVs of Nafion 1 wt %/GNPs 0.5 wt % (5 LbL) layers films at increased concentration of caffeine, recorded in 0.1 M NaCl supporting electrolyte (pH 2); (**B**) Plot of the peak currents vs. concentration of caffeine. Dashed line corresponds to a blank DPV measurement.

The as-prepared Nafion/GNPs LbL films showed a linear response in the range 50 μM‒5 mM with a limit of detection (LoD) calculated as 24 μM using cyclic voltammetry. The sensitivity of Nafion/GNPs LbL films can be further improved by using differential pulse voltammetry (DPV). Figure 8 shows the DPV curves recorded at different concentrations of caffeine from 20 µM to 0.5 mM. In this case, the oxidation peak occurs at 1.34 V.

**Scheme 2:**
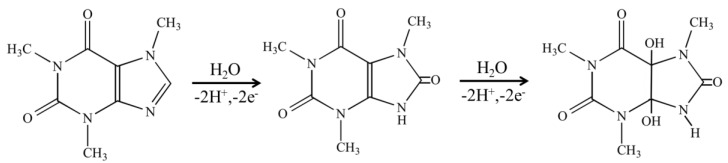
Mechanism of electrochemical oxidation of caffeine [57].

By using DPV, it is possible to further improve the sensitivity and the limit of detection. Figure 8A illustrates the DPV obtained at different concentrations of caffeine from 20 to 500 µM. The plot of the peak current vs. caffeine concentration (Figure 8B) exhibits a linear response from 20 µM to 0.25 mM with a regression equation expressed as *I_p_* (µA) = 0.138 [55] (µM) + 0.032 (*R^2^* = 0.995). The calculated detection limit LoD using the standard addition method was 0.032 µM [58]. The performance of Nafion/GNPs LbL film modified electrodes using cyclic voltammetry shows high linear ranges (Figure 7D), even though they are not the best detection limits when compared to other systems reported in literature (Table 2). Nonetheless, it is useful to emphasize that the aim of this work is the detection of caffeine in commercial drinks without dilution of the sample, where, typically, the concentration of caffeine is higher than 0.1 mM. The electrochemical oxidation mechanism of the caffeine is shown in Scheme 2. The electrochemical reaction of oxidation involves four electrons and four protons. The first step of the reaction is a two-electrons and two-proton oxidation of the C–8–N–9 bond giving the substituted uric acid. Then an immediate two-electron and two-proton oxidation of the 4,5-diol analogue of uric acid occurs, which rapidly fragmented [57,59].

### 3.5. Detection of Caffeine in Real Samples

**Figure 9:**
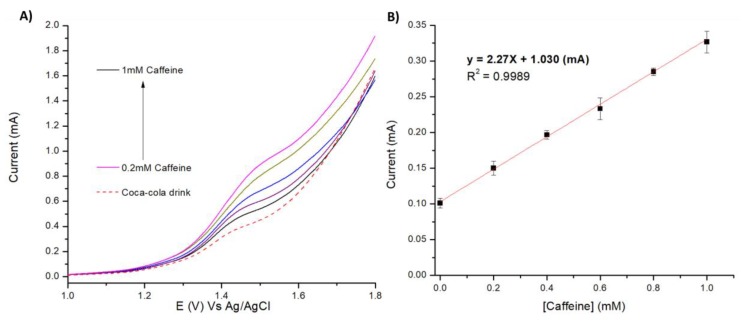
(**A**) Linear sweep voltammograms (LSVs) of Nafion/GNPs 5 LbL films recorded in Coca-Cola (dotted line) and after the addition of various concentrations of caffeine from 0.2 to 1 mM; supporting electrolyte, 0.1 M NaCl, and scan rate, 50 mV·s^−1^. (**B**) Plot of oxidation peak vs. concentration of caffeine for Nafion/GNPs 5 LbL films.

**Figure 10:**
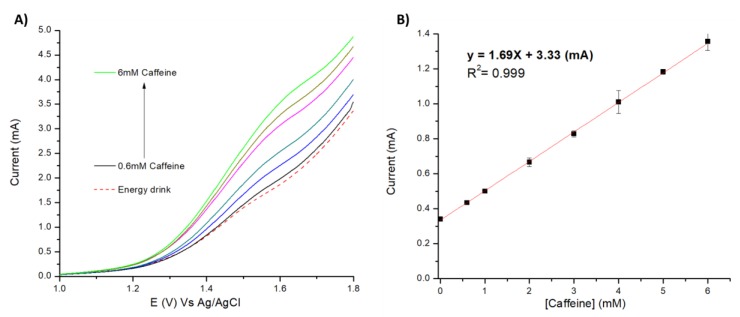
(**A**) LSVs of Nafion/GNPs 5 LbL films recorded in commercial energy drink (dotted line) and after the addition of various concentrations of caffeine from 0.6 mM to 6 mM; supporting electrolyte, 0.1 M NaCl, and scan rate, 50 mV·s^−1^. (**B**) Plot of oxidation peak vs. concentration of caffeine for Nafion/GNPs 5 LbL films.

The as-prepared Nafion/GNPs LbL films were utilized for the detection of caffeine in real samples, such as Coca-Cola and energy drink (Power Energy Shot). Both samples were analysed without any pre-treatment (no filtration, dilution or adjustment of pH) apart from the addition of 0.1 M NaCl as supporting electrolyte. The pH of the samples was determined to be 2.3 for Coca Cola and 2.7 for the power energy shot. These pH values are very similar in terms of the pH conditions utilized in Figure 7. Figure 9 and Figure 10 show the linear sweep voltammograms (LSVs) related to the Coca-Cola and energy drink, respectively, before and after standard addition of caffeine. The determination of caffeine in these samples was equal to 0.453 mM (0.088 mg/mL) for Coca-Cola and 1.97 mM (0.38 mg/mL) for the Power Energy Shot. Our results are in good agreement with the values obtained using standard spectrophotometric method and within the values of caffeine reported by the beverage producers on their respective websites (see Table 3) [60,61].

## 4. Conclusions

This work described for the first time a procedure for the fabrication of Nafion/graphite nanoplatelets films using a modified layer-by-layer method. By using synergistic interactions (hydrogen bonding and hydrophobic‒hydrophobic interactions), it is possible to build up multilayers of Nafion/GNPs using a very simple method that is suitable for the mass production of electrochemical sensors. The spectroscopic characterization of Nafion/GNPs LbL films showed the presence of oxygenated functionalities on the graphite surface that suggest the possibility of hydrogen bonding interactions with the sulfonic group of Nafion. These interactions, in combination with hydrophobic‒hydrophobic interactions between the Teflon backbone of Nafion and the carbon of graphite, drive the assembly mechanism. Nafion/GNPs LbL films were deposited on glassy carbon substrates and the as-prepared modified electrodes were evaluated for preliminary tests of detection of caffeine in fizzy beverages. The as-prepared Nafion/GNPs films showed the possibility of detecting caffeine with a limit of detection of 0.032 μM and a wide linear range (20‒250 μM) using differential pulse voltammetry. These values are comparable to the values reported in the literature (see Table 2). However, using cyclic voltammetry the LoD and linear range was found to be 24 μM and 50‒5000 μM. Detection of caffeine in real samples using CV is preferable because of the wider linear range, which allows for detection without dilution of the samples. For analysis of real samples where the concentration of caffeine is low, such as in drug formulations, DPV is the most suitable technique. Also, the possibility of fabricating Nafion/GNPs LbL films in an automated fashion is very appealing in view of mass production of electrochemical devices. Further studies are in progress to study in more detail the mechanism of self-assembly.

## Figures and Tables

**Table 1:** Parameters extracted using CV relating to five and 10 LbL layers of Nafion/GNPs. The experiments were performed in 0.1 M NaCl as a supporting electrolyte.

No. of Layers	*Φ*/(10^−5^) cm	Surface Coverage (*Γ*)/(10^−9^) mol/cm^2^	*C*_p_/(mol dm^−3^)	*D*_app_/(10^−10^ cm^2^·s^−1^ )
5	3 ± 1.7	1.65 ± 0.2	0.055 ± 0.02	1.06 ± 0.9
10	5 ± 1.9	2.80 ± 0.3	0.056 ± 0.02	1.47 ± 1.1

**Table 2:** Comparison of different modified electrodes for the determination of caffeine as reported in the latest literature.

	Electrochemical Technique	Linear Range (µM)	LOD (µM)	Reference
Functionalized MWCNT/GCE	DPV	10.0‒100.0	3.54	[62]
Nafion/Gr/GCE	DPV	0.4‒40.0 and 40‒600	0.12	[54]
PST/Nafion/GCE	LSV	0.3‒100.0	0.10	[63]
MWCNT-Nafion/GCE	DPV	2.945‒377.0	0.513	[64]
Large mesoporous carbon/Nafion/GCE	DPV	1.3‒230.0	0.47	[65]
MIP/Carbon Paste	DPV	0.06‒25.0	0.02	[66]
CA-Zn/GCE	DPV	39.8‒458	28.5	[67]
Fe_2_O_3_/PEDOT/rGO/GCE	DPV	1‒800	0.33	[68]
PLCY/N-CNT/GCE	DPV	0.4–140.0	0.02	[69]
PDA/AuNPs/GCE	DPV	100‒7500	0.79	[70]
AuNP-GCPE	DPV	25–150 and 200–1000	0.96	[71]
Poly(FA)/ GR /GCE	DPV	1‒160	0.08	[72]
Nafion/PDDA-MWCNT/GCE	DPV	0.3–80	0.05	[73]
Nafion/GNPs/LbL film	CV	50–5000	24	This work
Nafion/GNPs/LbL film	DPV	20‒250	0.03	This work

**Table 3:** Analysis of caffeine in real samples by the UV-Vis spectroscopy and Nafion/GNPs 5 LbL films coated electrode (*n* = 3).

Sample	Caffeine (mM)/(mg/mL)	
	Nafion/GNPs	UV-vis Spectroscopic ^1^
Coca-Cola	0.453 mM/0.0879 g·L^−1^	0.45 mM/0.088 g·L^−1^
Energy drink	1.97 mM/0.38 g·L^−1^	1.90 mM/0.37 g·L^−1^

^1^ Measured with UV-Vis absorption spectroscopy at 275 nm.

## Supplementary Materials

The following are available online at http://www.mdpi.com/2079-4991/9/2/221/s1, Figure S1: Video showing the procedure for the fabrication of Nafion/GNPs LbL films, Figure S2: Optical image of Nafion/GNPs films (5 and 10 layers) at 0.8% wt of GNPs, Figure S3: Optical image of Nafion/GNPs LbL films (left) and pristine GNP LbL films (right), Figure S4: CV of bare GCE (black line) and 5 Nafion/GNPs LbL film (green line) recorded in 0.1 M NaCl supporting electrolyte and 5 mM Ru[(NH_3_)]_6_^3+^; scan rates 50 mV s^−1^, Figure S5: Chronocoulometric plot of bare GCE (black line), 5 Nafion/GNPs LbL films (blue line) and 10 Nafion/GNPs LbL films (red line) recorded in 0.1M NaCl containing 5mM Ru[(NH_3_)]_6_^3+^, Figure S6: Profilometer patterns of Nafion/GNPs LbL films, Figure S7: CVs of bare glassy carbon recorded in 0.1 M NaCl supporting electrolyte (pH 2) containing 5 mM caffeine; scan rates 50 mV s^−1^, Figure S8: Protonated chemical structures of caffeine, Figure S9: CVs of 5 Nafion/GNPs LbL films recorded in 0.1 M NaCl supporting electrolyte (pH 2) at different concentrations of caffeine from 0.1 mM to 5 mM; scan rate, 50 mV s^−1^.
