# Supplementary material for: Voltammetric Detection of Caffeine in Beverages at Nafion/Graphite Nanoplatelets Layer-by-Layer Films
Source: Nanomaterials (Basel). 2019 Feb 7;9(2):221. doi: 10.3390/nano9020221 (PMC6410159; doi:10.3390/nano9020221)
Supplement: Supplementary file 1 [file nanomaterials-09-00221-s001.pdf]

Electronic Supporting information (ESI)

Voltammetric Detection of Caffeine in Beverages at Nafion/Graphite Nanoplatelets Layer-by-Layer Films

Sandra Hernandez-Aldave [1], Afshin Tarat [2], James D. McGettrick [3] and Paolo Bertoncello [1,4,*]

Synthesis process.mp4

Figure S1. Video showing the procedure for the fabrication of Nafion/GNPs LbL films.

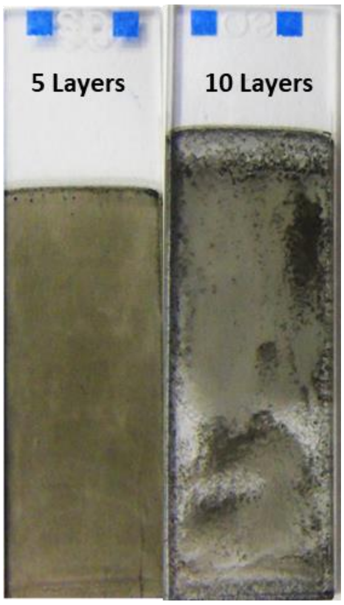

Figure S2. Optical image of Nafion/GNPs films (5 and 10 layers) at 0.8% wt of GNPs.

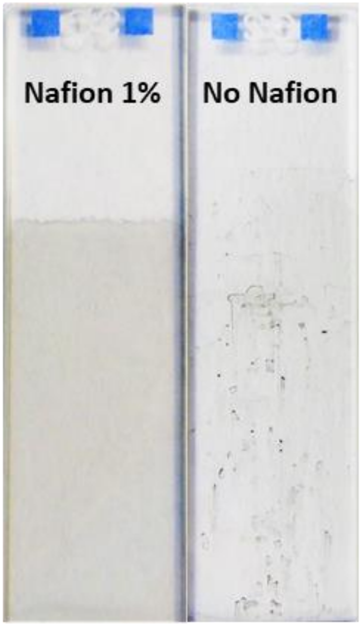

Figure S3. Optical image of Nafion/GNPs LbL films (left) and pristine GNP LbL films (right).

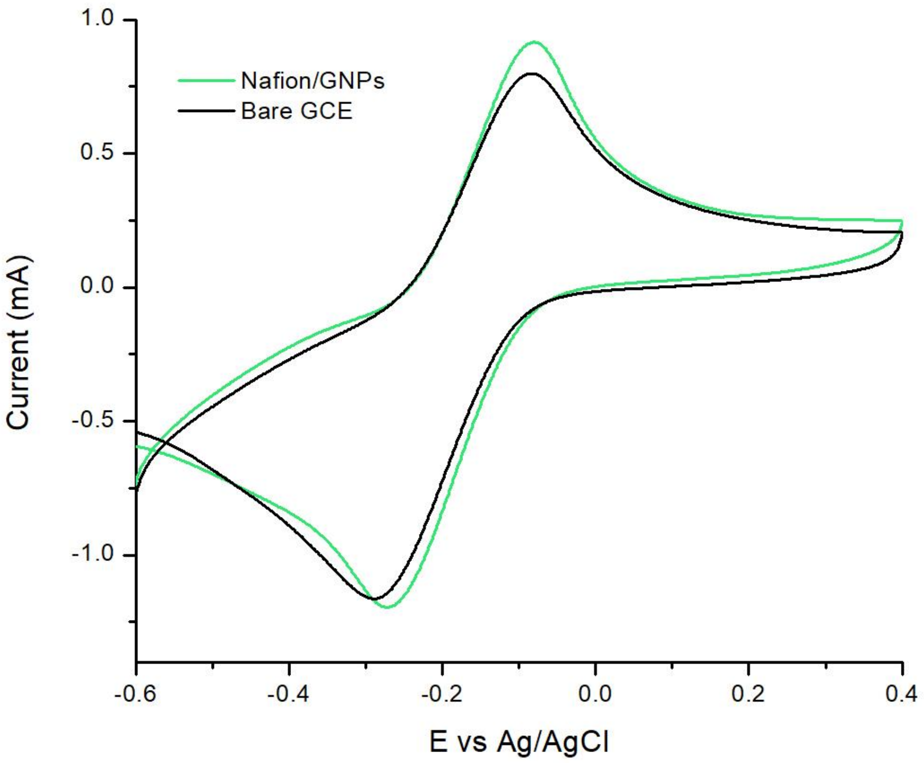

Figure S4. CV of bare GCE (black line) and 5 Nafion/GNPs LbL film (green line) recorded in 0.1 M NaCl supporting electrolyte and 5 mM $Ru[(NH_3)]_6^{3+}$; scan rates 50 mV s^{-1}.

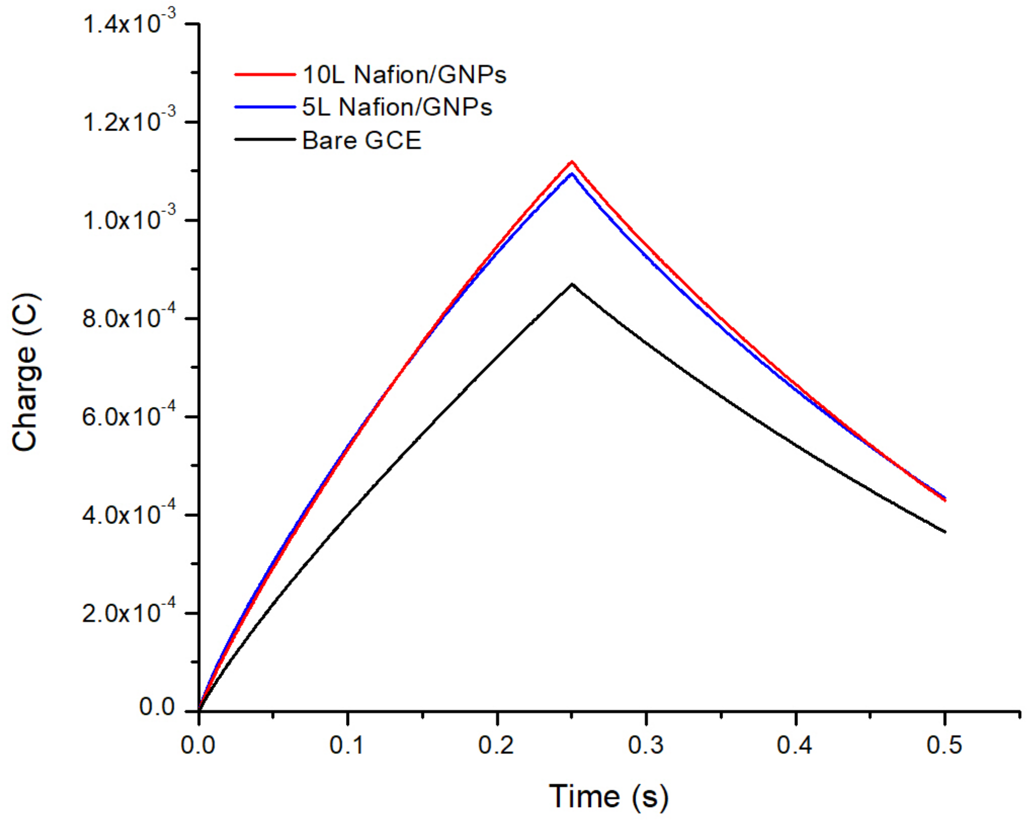

Figure S5. Chronocoulometric plot of bare GCE (black line), 5 Nafion/GNPs LbL films (blue line) and 10 Nafion/GNPs LbL films (red line) recorded in 0.1M NaCl containing 5mM $Ru[(NH_3)]_6^{3+}$.

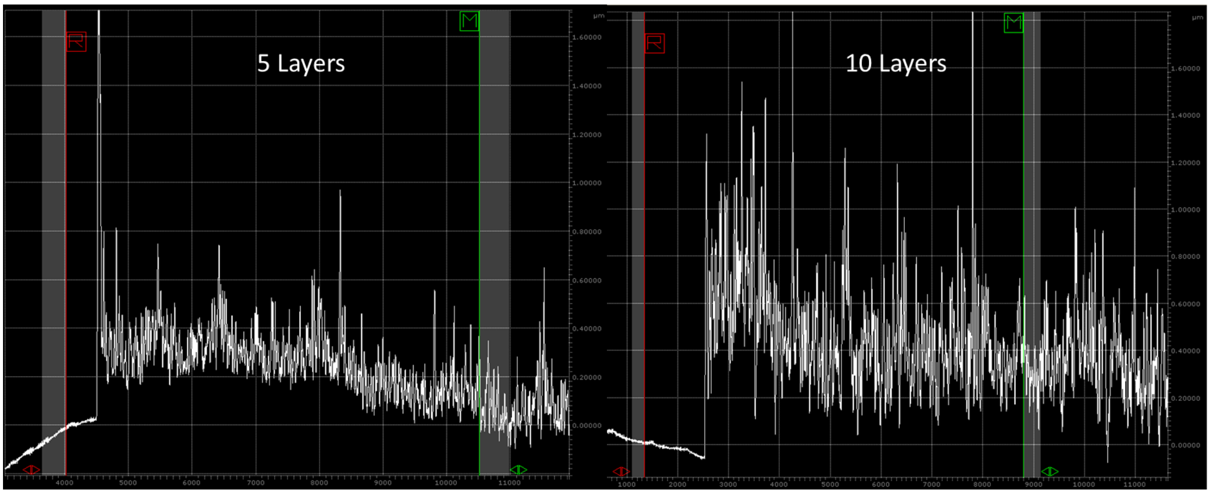

Figure S6. Profilometer patterns of Nafion/GNPs LbL films.

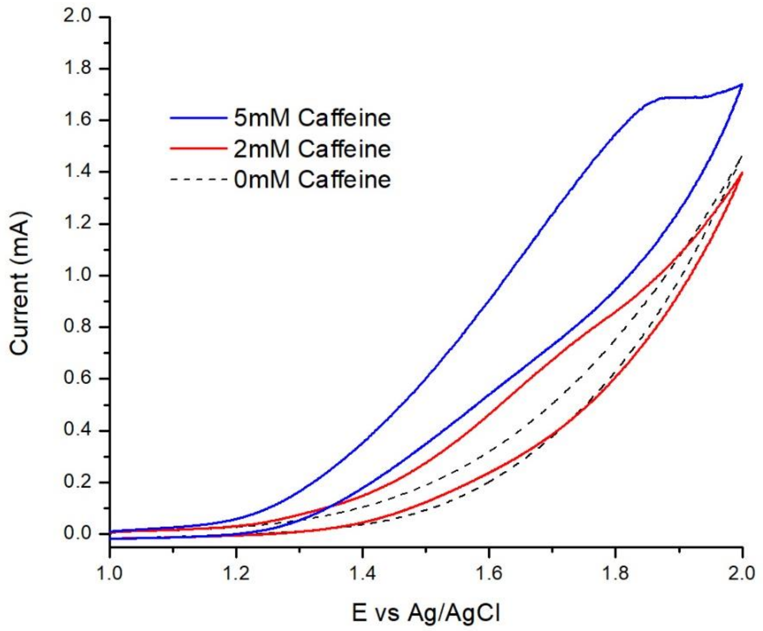

Figure S7. CVs of bare glassy carbon recorded in 0.1 M NaCl supporting electrolyte (pH 2) containing 5 mM caffeine; scan rates 50 mV s^{-1}.

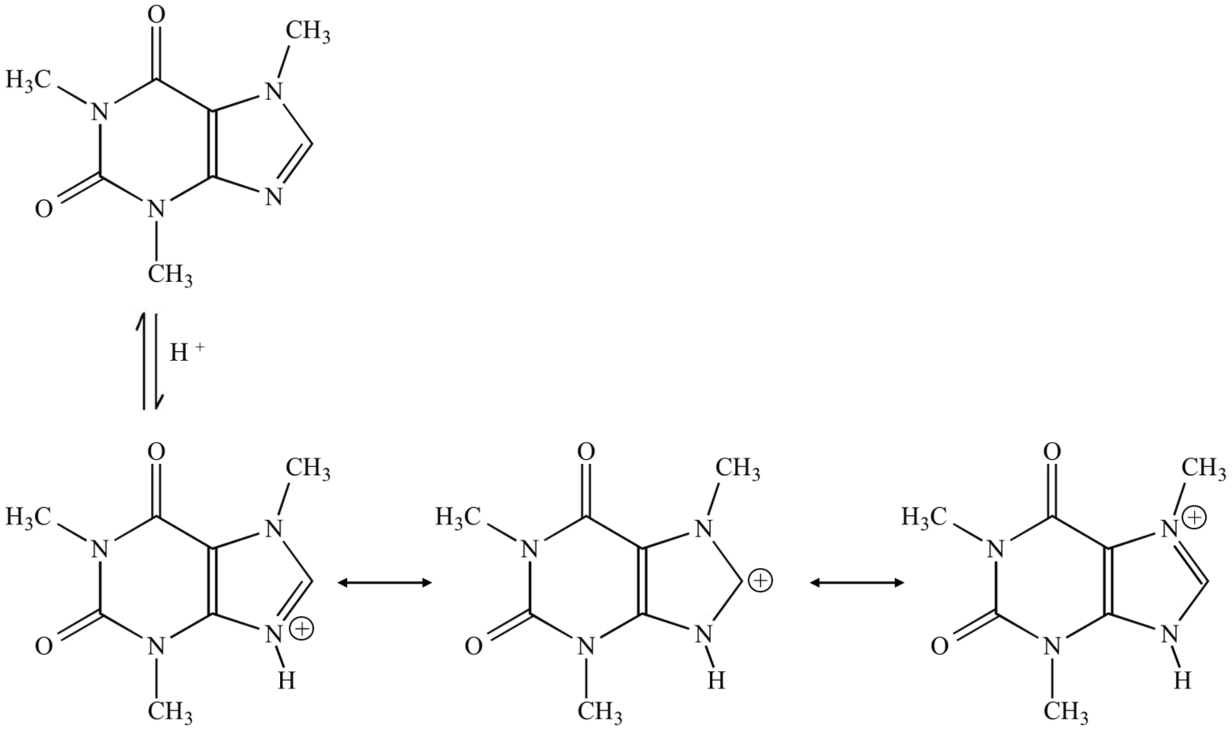

Figure S8. Protonated chemical structures of caffeine [1,2].

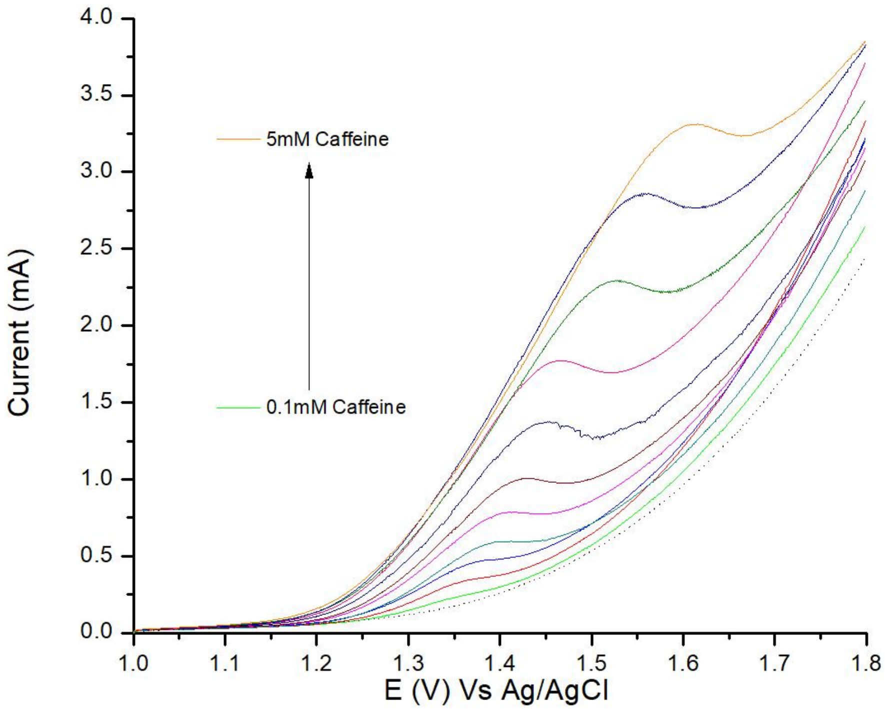

Figure S9. CVs of 5 Nafion/GNPs LbL films recorded in 0.1 M NaCl supporting electrolyte (pH 2) at different concentrations of caffeine from 0.1 mM to 5 mM; scan rate, 50 mV s^{-1}.

References

1. Bahrami, H.; Tabrizchi, M.; Farrokhpour, H. Protonation of caffeine: A theoretical and experimental study. *Chemical Physics* **2013**, *415*, 222-227, doi:https://doi.org/10.1016/j.chemphys.2013.01.022.
2. Oestreich-Janzen, S. Chemistry of Coffee, Change History: March 2013. S Oestreich-Janzen has updated the text throughout in this chapter. In *Reference Module in Chemistry, Molecular Sciences and Chemical Engineering*, Elsevier: 2013; https://doi.org/10.1016/B978-0-12-409547-2.02786-4.
